# 3,3′-Diallyl-1,1′-[*o*-phenyl­enebis(methyl­ene)]diimidazol-3-ium bis­(hexa­fluoro­phosphate)

**DOI:** 10.1107/S160053681103474X

**Published:** 2011-08-31

**Authors:** Rosenani A. Haque, Mohammed Z. Ghdhayeb, Madhukar Hemamalini, Hoong-Kun Fun

**Affiliations:** a School of Chemical Sciences, Universiti Sains Malaysia, 11800 USM, Penang, Malaysia; b X-ray Crystallography Unit, School of Physics, Universiti Sains Malaysia, 11800 USM, Penang, Malaysia

## Abstract

In the cation of the title mol­ecular salt, C_20_H_24_N_4_^2+^·2PF_6_^−^, the central benzene ring makes dihedral angles of 84.19 (7) and 79.10 (7)° with the pendant imidazole rings. In one of the hexa­fluoro­phosphate anions, the six F atoms are disordered over two sets of sites, with an occupancy ratio of 0.842 (3):0.158 (3). In the crystal, the cations and anions are linked by numerous C—H⋯F hydrogen bonds, thereby forming a three-dimensional network.

## Related literature

For applications and properties of *N*-heterocyclic carbenes, see: Bielawski & Grubbs (2000 ▶); Herrmann *et al.* (1998 ▶); Yeung *et al.* (2011 ▶); Jokic *et al.* (2010 ▶); Yu *et al.* (2010 ▶); Esteruelas *et al.* (2003 ▶). For the stability of the temperature controller used in the data collection, see: Cosier & Glazer (1986 ▶).
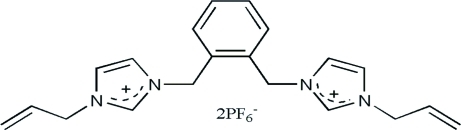


## Experimental

### 

#### Crystal data

C_20_H_24_N_4_^2+^·2PF_6_^−^
*M* *_r_* = 610.37
Triclinic, 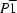

*a* = 7.3151 (3) Å
*b* = 12.4913 (4) Å
*c* = 13.8569 (5) Å
α = 101.810 (1)°
β = 94.603 (1)°
γ = 91.424 (1)°
*V* = 1234.27 (8) Å^3^
*Z* = 2
Mo *K*α radiation
μ = 0.29 mm^−1^
*T* = 100 K
0.82 × 0.61 × 0.48 mm

#### Data collection

Bruker APEXII DUO CCD diffractometer
Absorption correction: multi-scan (*SADABS*; Bruker, 2009 ▶) *T*_min_ = 0.801, *T*_max_ = 0.874
38348 measured reflections
10789 independent reflections
9422 reflections with *I* > 2σ(*I*)
*R*_int_ = 0.016

#### Refinement

*R*[*F* ^2^ > 2σ(*F* ^2^)] = 0.049
*wR*(*F* ^2^) = 0.142
*S* = 1.05
10789 reflections
368 parameters
15 restraints
H-atom parameters constrained
Δρ_max_ = 1.45 e Å^−3^
Δρ_min_ = −0.91 e Å^−3^

### 

Data collection: *APEX2* (Bruker, 2009 ▶); cell refinement: *SAINT* (Bruker, 2009 ▶); data reduction: *SAINT*; program(s) used to solve structure: *SHELXTL* (Sheldrick, 2008 ▶); program(s) used to refine structure: *SHELXTL*; molecular graphics: *SHELXTL*; software used to prepare material for publication: *SHELXTL* and *PLATON* (Spek, 2009 ▶).

## Supplementary Material

Crystal structure: contains datablock(s) global, I. DOI: 10.1107/S160053681103474X/hb6386sup1.cif

Structure factors: contains datablock(s) I. DOI: 10.1107/S160053681103474X/hb6386Isup2.hkl

Supplementary material file. DOI: 10.1107/S160053681103474X/hb6386Isup3.cml

Additional supplementary materials:  crystallographic information; 3D view; checkCIF report

## Figures and Tables

**Table 1:** Hydrogen-bond geometry (Å, °)

*D*—H⋯*A*	*D*—H	H⋯*A*	*D*⋯*A*	*D*—H⋯*A*
C4—H4*A*⋯F5^i^	0.93	2.44	3.2625 (17)	148
C9—H9*A*⋯F8*A*^ii^	0.93	2.38	3.303 (3)	171
C10—H10*A*⋯F2^iii^	0.93	2.47	3.2429 (18)	141
C14—H14*A*⋯F3	0.97	2.41	3.3065 (16)	154
C14—H14*B*⋯F9*A*^ii^	0.97	2.42	3.224 (2)	140
C15—H15*A*⋯F5	0.93	2.51	3.2100 (15)	132
C17—H17*A*⋯F12*A*^iv^	0.93	2.42	3.279 (2)	154
C18—H18*B*⋯F1^ii^	0.97	2.55	3.349 (2)	140
C19—H19*A*⋯F12*A*^v^	0.93	2.50	3.364 (2)	155
C20—H20*A*⋯F8*A*^vi^	0.93	2.40	3.158 (3)	139
C20—H20*B*⋯F1^vii^	0.93	2.45	3.267 (2)	146

Symmetry codes: (i) 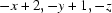; (ii) 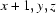; (iii) 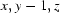; (iv) 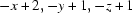; (v) 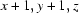; (vi) 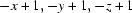; (vii) 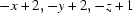.

**Fig. 1.**
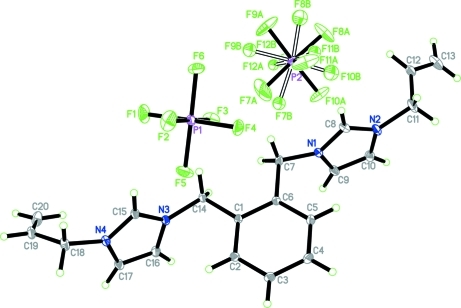
The asymmetric unit of the title compound, showing 30% probability displacement ellipsoids.

**Fig. 2.**
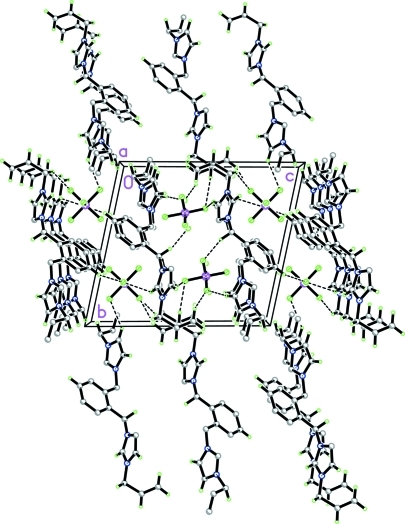
The crystal packing of the major component of the title compound, showing hydrogen-bonded (dashed lines) network.

## supplementary crystallographic information

### Comment

*N*-heterocyclic carbene (NHC) ligands have long been at the forefront of catalytic research and are studied extensively for the preparation of various transition metal catalysts (Bielawski & Grubbs, 2000; Herrmann *et al.*, 1998). NHCs, on account of their strong chelation, stability towards air and moisture, modest cost and their availability in ionic form, makes them versatile precursors in catalysis ranging from C–C coupling to olefin polymerizations (Yeung *et al.*, 2011; Jokic *et al.*, 2010; Yu *et al.*, 2010). The main point of focus in the reported compound is the presence of ally and methylene groups, which rotate the molecule when treated with metal ion to form a cage-like- structure suitable for the polymerization of ethylene and other higher olefins (Esteruelas *et al.*, 2003). Fortified by the highly active and interesting characteristics obtained from complexes ligated by bis- carbene NHC backbone, the present ligand system is designed and synthesized in view of getting stable and active olefin polymerization catalyst.

The asymmetric unit of the title compound, (Fig. 1), consists of a 1,2-bis(allylimidazole-1-ylmethyl)benzene cation and two hexafluoro phosphate anions. In one of the PF_6_^-^ octahedral anions, all F atoms are disordered over two sets of sites, with occupancy ratio of 0.842 (3): 0.158 (3). The central benzene (C1–C6) ring makes dihedral angles of 84.19 (7)° and 79.10 (7)° with the terminal imidazole (N1,N2/C8–C10)/ (N3,N4/C15–C17) rings, respectively.  The distorted octahedral geometry of phosphate ions has typical P–F distances [1.480 (9)–1.615 (7) Å] and F—P—F angles [47.5 (4)–179.59 (7)°].  All bond lengths and bond angles in (I) are in the range of expected values.

In the crystal (Fig. 2), the cations and anions are linked together *via* intermolecular C—H···F (Table 1) hydrogen bonds forming a three-dimensional network.

### Experimental

A mixture of imidazole (0.9 g, 13.0 mmol) and sodium hydroxide (0.5 g, 12 mmol) in DMSO (5 mL) was heated to 90°C for 2 h, and then was cooled to room temperature. A solution of 1,2-bis(bromomethyl)benzene  (1.5 g, 5.7 mmol)  in DMSO (10 mL) was added to the mixture and heated slowly to 40°C for 1 h with constant stirring. The solution obtained was poured into ice-cold water (40 mL). The precipitate was collected, washed with water, and recrystallized from methanol/water to give 1,2-bis(N-imidazole- 1-ylmethyl)benzene [1]  as a white solid (0.95 g, 79 %). Furthernore, a mixture of [1] (0.5 g, 2.1 mmol) and allyl bromide (0.7 g, 6.1 mmol) in acetonitrile (20 mL) was refluxed for 24 h. The solvent was removed under reduced pressure to yield a pale-brown oil, which was converted directly to its corresponding hexafluorophosphate salt by metathesis reaction using KPF_6_ (0.76g, 4.0 mmol) in 20 ml of methanol. The precipitate formed was collected and washed with distilled water (2 × 5 ml), and recrystallized from acetonitrile to give colorless solid. (1.1 g, 80 %). Colourless blocks of (I) were obtained by slow evaporation of the salt solution in acetonitrile at room temperature.

### Refinement

All hydrogen atoms were positioned geometrically [C–H = 0.93–0.97 Å] and were refined using a riding model, with *U*_iso_(H) = 1.2 *U*_eq_(C).  A rotating group model was applied to the methyl groups. In one of the PF_6_^-^ octahedra, all F atoms are disordered over two sets of sites, with occupancy ratio of 0.842 (3):0.158 (3).

### Crystal data

**Table:**

C_20_H_24_N_4_^2+^·2PF_6_^−^	*Z* = 2
*M**_r_* = 610.37	*F*(000) = 620
Triclinic, *P*1	*D*_x_ = 1.642 Mg m^−^^3^
Hall symbol: -P 1	Mo *K*α radiation, λ = 0.71073 Å
*a* = 7.3151 (3) Å	Cell parameters from 9808 reflections
*b* = 12.4913 (4) Å	θ = 2.5–35.1°
*c* = 13.8569 (5) Å	µ = 0.29 mm^−^^1^
α = 101.810 (1)°	*T* = 100 K
β = 94.603 (1)°	Block, colourless
γ = 91.424 (1)°	0.82 × 0.61 × 0.48 mm
*V* = 1234.27 (8) Å^3^	

### Data collection

**Table:**

Bruker APEXII DUO CCD diffractometer	10789 independent reflections
Radiation source: fine-focus sealed tube	9422 reflections with *I* > 2σ(*I*)
graphite	*R*_int_ = 0.016
φ and ω scans	θ_max_ = 35.1°, θ_min_ = 2.5°
Absorption correction: multi-scan (*SADABS*; Bruker, 2009)	*h* = −11→10
*T*_min_ = 0.801, *T*_max_ = 0.874	*k* = −20→20
38348 measured reflections	*l* = −22→22

### Refinement

**Table:**

Refinement on *F*^2^	Primary atom site location: structure-invariant direct methods
Least-squares matrix: full	Secondary atom site location: difference Fourier map
*R*[*F*^2^ > 2σ(*F*^2^)] = 0.049	Hydrogen site location: inferred from neighbouring sites
*wR*(*F*^2^) = 0.142	H-atom parameters constrained
*S* = 1.05	*w* = 1/[σ^2^(*F*_o_^2^) + (0.0703*P*)^2^ + 0.8687*P*] where *P* = (*F*_o_^2^ + 2*F*_c_^2^)/3
10789 reflections	(Δ/σ)_max_ = 0.001
368 parameters	Δρ_max_ = 1.45 e Å^−^^3^
15 restraints	Δρ_min_ = −0.91 e Å^−^^3^

### Special details

**Table:**

Experimental. The crystal was placed in the cold stream of an Oxford Cryosystems Cobra open-flow nitrogen cryostat (Cosier & Glazer, 1986) operating at 100.0 (1) K.
Geometry. All s.u.'s (except the s.u. in the dihedral angle between two l.s. planes) are estimated using the full covariance matrix. The cell s.u.'s are taken into account individually in the estimation of s.u.'s in distances, angles and torsion angles; correlations between s.u.'s in cell parameters are only used when they are defined by crystal symmetry. An approximate (isotropic) treatment of cell s.u.'s is used for estimating s.u.'s involving l.s. planes.
Refinement. Refinement of F^2^ against ALL reflections. The weighted R-factor wR and goodness of fit S are based on F^2^, conventional R-factors R are based on F, with F set to zero for negative F^2^. The threshold expression of F^2^ > 2σ(F^2^) is used only for calculating R-factors(gt) etc. and is not relevant to the choice of reflections for refinement. R-factors based on F^2^ are statistically about twice as large as those based on F, and R- factors based on ALL data will be even larger.

### Fractional atomic coordinates and isotropic or equivalent isotropic displacement parameters (Å^2^)

**Table:**

	*x*	*y*	*z*	*U*_iso_*/*U*_eq_	Occ. (<1)
P1	0.54544 (4)	0.74563 (2)	0.15374 (2)	0.01863 (7)	
F1	0.5790 (2)	0.83688 (11)	0.25409 (9)	0.0502 (3)	
F2	0.5502 (2)	0.84110 (9)	0.09108 (10)	0.0468 (3)	
F3	0.53920 (14)	0.65162 (9)	0.21542 (9)	0.0359 (2)	
F4	0.51464 (15)	0.65782 (8)	0.05282 (7)	0.0340 (2)	
F5	0.76397 (14)	0.73551 (11)	0.15226 (8)	0.0413 (3)	
F6	0.32943 (13)	0.75680 (9)	0.15780 (9)	0.0344 (2)	
P2	0.22213 (5)	0.30131 (3)	0.40480 (2)	0.01929 (7)	
F7A	0.3956 (4)	0.3793 (2)	0.4200 (2)	0.0999 (10)	0.842 (3)
F8A	0.0484 (3)	0.2183 (2)	0.3855 (2)	0.0850 (9)	0.842 (3)
F9A	0.0911 (3)	0.39801 (16)	0.43888 (12)	0.0695 (8)	0.842 (3)
F10A	0.3494 (3)	0.20165 (14)	0.36918 (11)	0.0528 (5)	0.842 (3)
F11A	0.2050 (4)	0.3261 (3)	0.29691 (16)	0.0730 (9)	0.842 (3)
F12A	0.2420 (3)	0.27306 (15)	0.51330 (10)	0.0334 (3)	0.842 (3)
F7B	0.4422 (10)	0.3158 (7)	0.4133 (5)	0.0361 (16)*	0.158 (3)
F8B	0.0083 (12)	0.3055 (8)	0.4068 (7)	0.052 (2)*	0.158 (3)
F9B	0.2255 (12)	0.4261 (6)	0.4665 (6)	0.0400 (17)*	0.158 (3)
F10B	0.2296 (15)	0.1851 (7)	0.3547 (7)	0.052 (2)*	0.158 (3)
F11B	0.1483 (12)	0.3031 (7)	0.2934 (7)	0.0314 (17)*	0.158 (3)
F12B	0.2737 (12)	0.3018 (7)	0.5120 (6)	0.0256 (17)*	0.158 (3)
N1	0.59431 (14)	0.27887 (8)	0.20617 (8)	0.01726 (16)	
N2	0.42044 (15)	0.13849 (9)	0.13427 (8)	0.02092 (19)	
N3	1.06450 (14)	0.66033 (8)	0.35317 (7)	0.01654 (16)	
N4	1.20454 (14)	0.81979 (8)	0.38440 (8)	0.01757 (17)	
C1	0.95950 (15)	0.49943 (8)	0.22093 (8)	0.01440 (16)	
C2	1.10935 (16)	0.51829 (9)	0.16968 (9)	0.01721 (18)	
H2A	1.1900	0.5779	0.1953	0.021*	
C3	1.13981 (17)	0.44894 (10)	0.08057 (9)	0.01942 (19)	
H3A	1.2403	0.4622	0.0470	0.023*	
C4	1.01961 (18)	0.35991 (10)	0.04209 (9)	0.0199 (2)	
H4A	1.0396	0.3132	−0.0173	0.024*	
C5	0.86910 (17)	0.34065 (10)	0.09256 (9)	0.01891 (19)	
H5A	0.7886	0.2811	0.0665	0.023*	
C6	0.83773 (15)	0.40962 (9)	0.18177 (8)	0.01586 (17)	
C7	0.67134 (19)	0.39112 (10)	0.23602 (11)	0.0239 (2)	
H7A	0.7062	0.4070	0.3067	0.029*	
H7B	0.5780	0.4414	0.2229	0.029*	
C8	0.44306 (16)	0.24647 (11)	0.14644 (9)	0.0204 (2)	
H8A	0.3660	0.2917	0.1179	0.024*	
C9	0.67130 (17)	0.18858 (10)	0.23291 (10)	0.0215 (2)	
H9A	0.7785	0.1880	0.2737	0.026*	
C10	0.56075 (19)	0.10063 (10)	0.18845 (11)	0.0238 (2)	
H10A	0.5769	0.0284	0.1937	0.029*	
C11	0.2661 (2)	0.07107 (15)	0.07509 (11)	0.0331 (3)	
H11A	0.3100	0.0005	0.0449	0.040*	
H11B	0.2206	0.1065	0.0224	0.040*	
C12	0.11325 (18)	0.05405 (12)	0.13527 (10)	0.0243 (2)	
H12A	0.0634	0.1156	0.1718	0.029*	
C13	0.0442 (2)	−0.04225 (15)	0.14014 (14)	0.0371 (4)	
H13A	0.0912	−0.1053	0.1044	0.044*	
H13B	−0.0518	−0.0475	0.1793	0.044*	
C14	0.92288 (16)	0.57296 (9)	0.31803 (8)	0.01732 (18)	
H14A	0.8051	0.6056	0.3100	0.021*	
H14B	0.9152	0.5289	0.3678	0.021*	
C15	1.04896 (16)	0.76409 (9)	0.34527 (8)	0.01693 (18)	
H15A	0.9468	0.7930	0.3172	0.020*	
C16	1.23543 (18)	0.64893 (10)	0.39896 (10)	0.0215 (2)	
H16A	1.2816	0.5849	0.4138	0.026*	
C17	1.32333 (17)	0.74889 (10)	0.41826 (10)	0.0219 (2)	
H17A	1.4414	0.7663	0.4486	0.026*	
C18	1.24241 (19)	0.93714 (10)	0.39202 (10)	0.0224 (2)	
H18A	1.1445	0.9658	0.3545	0.027*	
H18B	1.3558	0.9472	0.3625	0.027*	
C19	1.25869 (19)	1.00104 (10)	0.49639 (10)	0.0232 (2)	
H19A	1.2934	1.0749	0.5061	0.028*	
C20	1.2287 (3)	0.96299 (12)	0.57563 (11)	0.0311 (3)	
H20A	1.1938	0.8897	0.5696	0.037*	
H20B	1.2425	1.0094	0.6377	0.037*	

### Atomic displacement parameters (Å^2^)

**Table:**

	*U*^11^	*U*^22^	*U*^33^	*U*^12^	*U*^13^	*U*^23^
P1	0.01926 (14)	0.01710 (13)	0.01805 (13)	−0.00355 (10)	0.00474 (10)	−0.00048 (10)
F1	0.0604 (8)	0.0465 (6)	0.0319 (5)	−0.0184 (6)	0.0144 (5)	−0.0209 (5)
F2	0.0717 (9)	0.0278 (5)	0.0478 (6)	−0.0017 (5)	0.0234 (6)	0.0169 (4)
F3	0.0299 (5)	0.0422 (5)	0.0423 (5)	0.0034 (4)	0.0019 (4)	0.0250 (5)
F4	0.0390 (5)	0.0303 (4)	0.0255 (4)	0.0027 (4)	−0.0032 (4)	−0.0085 (3)
F5	0.0190 (4)	0.0666 (8)	0.0344 (5)	−0.0085 (4)	0.0067 (3)	0.0012 (5)
F6	0.0226 (4)	0.0368 (5)	0.0476 (6)	0.0089 (3)	0.0096 (4)	0.0142 (4)
P2	0.01918 (14)	0.02183 (14)	0.01876 (13)	0.00482 (10)	0.00316 (10)	0.00761 (10)
F7A	0.0879 (18)	0.0702 (16)	0.138 (2)	−0.0555 (15)	0.0115 (16)	0.0189 (15)
F8A	0.0454 (10)	0.1067 (19)	0.0978 (17)	−0.0443 (12)	−0.0295 (10)	0.0286 (14)
F9A	0.1145 (19)	0.0675 (11)	0.0424 (8)	0.0716 (13)	0.0364 (10)	0.0293 (8)
F10A	0.0746 (12)	0.0617 (9)	0.0329 (6)	0.0502 (9)	0.0262 (7)	0.0210 (6)
F11A	0.0772 (16)	0.125 (2)	0.0434 (9)	0.0672 (16)	0.0339 (10)	0.0599 (12)
F12A	0.0513 (9)	0.0343 (8)	0.0182 (5)	0.0205 (7)	0.0089 (5)	0.0097 (5)
N1	0.0144 (4)	0.0159 (4)	0.0204 (4)	−0.0024 (3)	0.0010 (3)	0.0019 (3)
N2	0.0190 (4)	0.0244 (5)	0.0175 (4)	−0.0083 (3)	−0.0011 (3)	0.0021 (3)
N3	0.0161 (4)	0.0142 (4)	0.0177 (4)	−0.0004 (3)	−0.0010 (3)	0.0007 (3)
N4	0.0180 (4)	0.0157 (4)	0.0179 (4)	−0.0024 (3)	−0.0017 (3)	0.0025 (3)
C1	0.0146 (4)	0.0133 (4)	0.0151 (4)	0.0005 (3)	0.0011 (3)	0.0026 (3)
C2	0.0162 (4)	0.0161 (4)	0.0198 (4)	−0.0006 (3)	0.0029 (3)	0.0044 (3)
C3	0.0195 (5)	0.0205 (5)	0.0198 (5)	0.0027 (4)	0.0062 (4)	0.0057 (4)
C4	0.0228 (5)	0.0205 (5)	0.0160 (4)	0.0024 (4)	0.0045 (4)	0.0014 (4)
C5	0.0199 (5)	0.0177 (4)	0.0172 (4)	−0.0011 (4)	0.0021 (4)	−0.0008 (3)
C6	0.0156 (4)	0.0146 (4)	0.0165 (4)	−0.0011 (3)	0.0026 (3)	0.0009 (3)
C7	0.0235 (5)	0.0163 (4)	0.0297 (6)	−0.0052 (4)	0.0115 (5)	−0.0034 (4)
C8	0.0154 (4)	0.0248 (5)	0.0219 (5)	−0.0023 (4)	−0.0009 (4)	0.0086 (4)
C9	0.0177 (5)	0.0213 (5)	0.0251 (5)	−0.0005 (4)	−0.0033 (4)	0.0058 (4)
C10	0.0244 (6)	0.0176 (5)	0.0288 (6)	−0.0020 (4)	0.0016 (4)	0.0044 (4)
C11	0.0277 (7)	0.0465 (8)	0.0199 (5)	−0.0208 (6)	−0.0006 (5)	−0.0018 (5)
C12	0.0194 (5)	0.0271 (6)	0.0241 (5)	−0.0062 (4)	0.0011 (4)	0.0010 (4)
C13	0.0315 (7)	0.0406 (8)	0.0410 (8)	−0.0171 (6)	−0.0132 (6)	0.0212 (7)
C14	0.0175 (4)	0.0153 (4)	0.0174 (4)	−0.0026 (3)	0.0020 (3)	−0.0006 (3)
C15	0.0175 (4)	0.0155 (4)	0.0166 (4)	−0.0004 (3)	−0.0015 (3)	0.0018 (3)
C16	0.0202 (5)	0.0169 (4)	0.0253 (5)	0.0021 (4)	−0.0047 (4)	0.0020 (4)
C17	0.0177 (5)	0.0192 (5)	0.0262 (5)	−0.0002 (4)	−0.0051 (4)	0.0019 (4)
C18	0.0269 (6)	0.0175 (5)	0.0229 (5)	−0.0058 (4)	−0.0013 (4)	0.0065 (4)
C19	0.0243 (5)	0.0151 (4)	0.0276 (6)	−0.0024 (4)	−0.0049 (4)	0.0018 (4)
C20	0.0443 (8)	0.0246 (6)	0.0216 (5)	0.0020 (5)	−0.0021 (5)	0.0001 (4)

### Geometric parameters (Å, °)

**Table:**

P1—F4	1.5870 (9)	C2—H2A	0.9300
P1—F3	1.5893 (10)	C3—C4	1.3884 (18)
P1—F6	1.5942 (10)	C3—H3A	0.9300
P1—F1	1.6049 (10)	C4—C5	1.3923 (17)
P1—F5	1.6081 (11)	C4—H4A	0.9300
P1—F2	1.6137 (11)	C5—C6	1.3937 (16)
P2—F10B	1.480 (9)	C5—H5A	0.9300
P2—F12B	1.502 (9)	C6—C7	1.5179 (17)
P2—F7A	1.5544 (19)	C7—H7A	0.9700
P2—F8B	1.569 (9)	C7—H7B	0.9700
P2—F9A	1.5803 (14)	C8—H8A	0.9300
P2—F11A	1.5838 (16)	C9—C10	1.3562 (18)
P2—F8A	1.5897 (17)	C9—H9A	0.9300
P2—F10A	1.5935 (13)	C10—H10A	0.9300
P2—F11B	1.599 (10)	C11—C12	1.484 (2)
P2—F7B	1.607 (7)	C11—H11A	0.9700
P2—F12A	1.6095 (13)	C11—H11B	0.9700
P2—F9B	1.615 (7)	C12—C13	1.310 (2)
N1—C8	1.3292 (15)	C12—H12A	0.9300
N1—C9	1.3769 (16)	C13—H13A	0.9300
N1—C7	1.4630 (15)	C13—H13B	0.9300
N2—C8	1.3290 (17)	C14—H14A	0.9700
N2—C10	1.3735 (18)	C14—H14B	0.9700
N2—C11	1.4758 (17)	C15—H15A	0.9300
N3—C15	1.3297 (14)	C16—C17	1.3564 (17)
N3—C16	1.3802 (16)	C16—H16A	0.9300
N3—C14	1.4652 (15)	C17—H17A	0.9300
N4—C15	1.3343 (15)	C18—C19	1.4960 (19)
N4—C17	1.3793 (16)	C18—H18A	0.9700
N4—C18	1.4657 (16)	C18—H18B	0.9700
C1—C2	1.3936 (16)	C19—C20	1.314 (2)
C1—C6	1.4036 (15)	C19—H19A	0.9300
C1—C14	1.5132 (15)	C20—H20A	0.9300
C2—C3	1.3931 (17)	C20—H20B	0.9300
			
F4—P1—F3	90.88 (6)	C10—N2—C11	125.68 (13)
F4—P1—F6	90.95 (6)	C15—N3—C16	108.80 (10)
F3—P1—F6	89.82 (6)	C15—N3—C14	125.17 (10)
F4—P1—F1	178.35 (7)	C16—N3—C14	126.03 (10)
F3—P1—F1	90.66 (7)	C15—N4—C17	108.63 (10)
F6—P1—F1	89.64 (7)	C15—N4—C18	125.89 (11)
F4—P1—F5	90.27 (6)	C17—N4—C18	125.46 (10)
F3—P1—F5	89.73 (6)	C2—C1—C6	119.38 (10)
F6—P1—F5	178.71 (6)	C2—C1—C14	122.12 (10)
F1—P1—F5	89.15 (7)	C6—C1—C14	118.50 (10)
F4—P1—F2	89.10 (6)	C3—C2—C1	120.81 (11)
F3—P1—F2	179.59 (7)	C3—C2—H2A	119.6
F6—P1—F2	89.77 (7)	C1—C2—H2A	119.6
F1—P1—F2	89.37 (7)	C4—C3—C2	119.76 (11)
F5—P1—F2	90.68 (7)	C4—C3—H3A	120.1
F10B—P2—F12B	104.3 (5)	C2—C3—H3A	120.1
F10B—P2—F7A	120.8 (4)	C3—C4—C5	119.85 (11)
F12B—P2—F7A	81.8 (3)	C3—C4—H4A	120.1
F10B—P2—F8B	97.6 (6)	C5—C4—H4A	120.1
F12B—P2—F8B	99.3 (5)	C4—C5—C6	120.74 (11)
F7A—P2—F8B	140.3 (4)	C4—C5—H5A	119.6
F10B—P2—F9A	144.9 (4)	C6—C5—H5A	119.6
F12B—P2—F9A	88.4 (4)	C5—C6—C1	119.47 (10)
F7A—P2—F9A	93.11 (16)	C5—C6—C7	121.16 (10)
F8B—P2—F9A	47.5 (4)	C1—C6—C7	119.35 (10)
F10B—P2—F11A	85.7 (4)	N1—C7—C6	112.31 (10)
F12B—P2—F11A	165.4 (3)	N1—C7—H7A	109.1
F7A—P2—F11A	83.93 (18)	C6—C7—H7A	109.1
F8B—P2—F11A	89.8 (4)	N1—C7—H7B	109.1
F9A—P2—F11A	89.34 (9)	C6—C7—H7B	109.1
F10B—P2—F8A	57.0 (4)	H7A—C7—H7B	107.9
F12B—P2—F8A	99.6 (3)	N2—C8—N1	108.53 (11)
F7A—P2—F8A	177.58 (17)	N2—C8—H8A	125.7
F9A—P2—F8A	88.93 (15)	N1—C8—H8A	125.7
F11A—P2—F8A	94.79 (16)	C10—C9—N1	106.84 (11)
F12B—P2—F10A	92.3 (3)	C10—C9—H9A	126.6
F7A—P2—F10A	88.43 (15)	N1—C9—H9A	126.6
F8B—P2—F10A	130.9 (4)	C9—C10—N2	107.04 (11)
F9A—P2—F10A	178.41 (13)	C9—C10—H10A	126.5
F11A—P2—F10A	90.41 (9)	N2—C10—H10A	126.5
F8A—P2—F10A	89.52 (14)	N2—C11—C12	112.51 (11)
F10B—P2—F11B	77.5 (5)	N2—C11—H11A	109.1
F12B—P2—F11B	174.7 (5)	C12—C11—H11A	109.1
F7A—P2—F11B	101.7 (3)	N2—C11—H11B	109.1
F8B—P2—F11B	75.5 (5)	C12—C11—H11B	109.1
F9A—P2—F11B	87.4 (4)	H11A—C11—H11B	107.8
F8A—P2—F11B	77.1 (3)	C13—C12—C11	124.08 (16)
F10A—P2—F11B	91.9 (4)	C13—C12—H12A	118.0
F10B—P2—F7B	91.2 (5)	C11—C12—H12A	118.0
F12B—P2—F7B	77.3 (4)	C12—C13—H13A	120.0
F8B—P2—F7B	171.1 (5)	C12—C13—H13B	120.0
F9A—P2—F7B	123.7 (3)	H13A—C13—H13B	120.0
F11A—P2—F7B	92.0 (3)	N3—C14—C1	113.10 (9)
F8A—P2—F7B	146.7 (3)	N3—C14—H14A	109.0
F10A—P2—F7B	57.8 (3)	C1—C14—H14A	109.0
F11B—P2—F7B	107.7 (4)	N3—C14—H14B	109.0
F10B—P2—F12A	92.9 (4)	C1—C14—H14B	109.0
F7A—P2—F12A	96.31 (15)	H14A—C14—H14B	107.8
F8B—P2—F12A	91.0 (4)	N3—C15—N4	108.57 (10)
F9A—P2—F12A	92.16 (7)	N3—C15—H15A	125.7
F11A—P2—F12A	178.46 (10)	N4—C15—H15A	125.7
F8A—P2—F12A	84.92 (12)	C17—C16—N3	106.94 (11)
F10A—P2—F12A	88.08 (7)	C17—C16—H16A	126.5
F11B—P2—F12A	162.0 (3)	N3—C16—H16A	126.5
F7B—P2—F12A	87.4 (3)	C16—C17—N4	107.06 (10)
F10B—P2—F9B	175.4 (5)	C16—C17—H17A	126.5
F12B—P2—F9B	71.5 (4)	N4—C17—H17A	126.5
F7A—P2—F9B	57.3 (3)	N4—C18—C19	113.13 (10)
F8B—P2—F9B	85.1 (5)	N4—C18—H18A	109.0
F11A—P2—F9B	98.1 (3)	C19—C18—H18A	109.0
F8A—P2—F9B	125.0 (3)	N4—C18—H18B	109.0
F10A—P2—F9B	143.2 (3)	C19—C18—H18B	109.0
F11B—P2—F9B	106.8 (4)	H18A—C18—H18B	107.8
F7B—P2—F9B	86.0 (4)	C20—C19—C18	126.33 (12)
F12A—P2—F9B	83.3 (3)	C20—C19—H19A	116.8
C8—N1—C9	108.77 (10)	C18—C19—H19A	116.8
C8—N1—C7	126.00 (11)	C19—C20—H20A	120.0
C9—N1—C7	125.19 (11)	C19—C20—H20B	120.0
C8—N2—C10	108.81 (10)	H20A—C20—H20B	120.0
C8—N2—C11	125.47 (13)		
			
C6—C1—C2—C3	−0.28 (17)	C8—N2—C10—C9	−0.89 (16)
C14—C1—C2—C3	179.80 (11)	C11—N2—C10—C9	−178.88 (13)
C1—C2—C3—C4	0.05 (18)	C8—N2—C11—C12	−92.85 (18)
C2—C3—C4—C5	0.21 (18)	C10—N2—C11—C12	84.81 (19)
C3—C4—C5—C6	−0.24 (19)	N2—C11—C12—C13	−125.16 (17)
C4—C5—C6—C1	0.01 (18)	C15—N3—C14—C1	101.58 (13)
C4—C5—C6—C7	178.37 (12)	C16—N3—C14—C1	−77.92 (15)
C2—C1—C6—C5	0.25 (17)	C2—C1—C14—N3	−2.85 (15)
C14—C1—C6—C5	−179.82 (10)	C6—C1—C14—N3	177.22 (10)
C2—C1—C6—C7	−178.15 (11)	C16—N3—C15—N4	0.08 (14)
C14—C1—C6—C7	1.78 (16)	C14—N3—C15—N4	−179.49 (10)
C8—N1—C7—C6	−103.72 (15)	C17—N4—C15—N3	0.09 (14)
C9—N1—C7—C6	73.79 (16)	C18—N4—C15—N3	−178.87 (11)
C5—C6—C7—N1	20.64 (18)	C15—N3—C16—C17	−0.22 (15)
C1—C6—C7—N1	−161.00 (11)	C14—N3—C16—C17	179.35 (11)
C10—N2—C8—N1	0.45 (15)	N3—C16—C17—N4	0.26 (15)
C11—N2—C8—N1	178.44 (12)	C15—N4—C17—C16	−0.22 (15)
C9—N1—C8—N2	0.16 (15)	C18—N4—C17—C16	178.74 (12)
C7—N1—C8—N2	178.01 (11)	C15—N4—C18—C19	112.08 (14)
C8—N1—C9—C10	−0.71 (15)	C17—N4—C18—C19	−66.71 (17)
C7—N1—C9—C10	−178.59 (12)	N4—C18—C19—C20	−5.5 (2)
N1—C9—C10—N2	0.96 (15)		

### Hydrogen-bond geometry (Å, °)

**Table:**

*D*—H···*A*	*D*—H	H···*A*	*D*···*A*	*D*—H···*A*
C4—H4A···F5^i^	0.93	2.44	3.2625 (17)	148
C9—H9A···F8A^ii^	0.93	2.38	3.303 (3)	171
C10—H10A···F2^iii^	0.93	2.47	3.2429 (18)	141
C14—H14A···F3	0.97	2.41	3.3065 (16)	154
C14—H14B···F9A^ii^	0.97	2.42	3.224 (2)	140
C15—H15A···F5	0.93	2.51	3.2100 (15)	132
C17—H17A···F12A^iv^	0.93	2.42	3.279 (2)	154
C18—H18B···F1^ii^	0.97	2.55	3.349 (2)	140
C19—H19A···F12A^v^	0.93	2.50	3.364 (2)	155
C20—H20A···F8A^vi^	0.93	2.40	3.158 (3)	139
C20—H20B···F1^vii^	0.93	2.45	3.267 (2)	146

Symmetry codes: (i) −*x*+2, −*y*+1, −*z*; (ii) *x*+1, *y*, *z*; (iii) *x*, *y*−1, *z*; (iv) −*x*+2, −*y*+1, −*z*+1; (v) *x*+1, *y*+1, *z*; (vi) −*x*+1, −*y*+1, −*z*+1; (vii) −*x*+2, −*y*+2, −*z*+1.
